# MassARRAY analysis of twelve cancer related SNPs in esophageal squamous cell carcinoma in J&K, India

**DOI:** 10.1186/s12885-020-06991-2

**Published:** 2020-06-01

**Authors:** Ruchi Shah, Varun Sharma, Amrita Bhat, Hemender Singh, Indu Sharma, Sonali Verma, Gh. Rasool Bhat, Bhanu Sharma, Divya Bakshi, Rakesh Kumar, Nazir Ahmed Dar

**Affiliations:** 1 School of Biotechnology, Shri Mata Vaishno Devi University, Katra, Jammu and Kashmir 182320 India; 2 Human Genetics Research Group, Shri Mata Vaishno Devi University, Katra, Jammu and Kashmir 182320 India; 3 Cancer Genetics Research Group, Shri Mata Vaishno Devi University, Katra, Jammu and Kashmir 182320 India; 4 Department of Biochemistry, University of Kashmir, Srinagar, Jammu and Kashmir 180001 India

**Keywords:** Single nucleotide polymorphism, Esophageal cancer, Jammu and Kashmir

## Abstract

**Background:**

MassARRAY (Agena Bioscience™) combines competitive PCR with MALDI-TOF mass spectrometry (MS) analysis that gives highly accurate, sensitive, and high-throughput methods for the quantitative analysis of variation of gene expression in multiple samples. SNPs (Single Nucleotide Polymorphisms) have a very high potential of discovering disease-gene relationships. SNP-genotyping through MassARRAY is not only a cost-effective genotyping method but also provides a platform to validate variants observed through a high-throughput Next-generation sequencing (NGS).

**Methods:**

In the present study, we have incorporated the use of matrix-assisted laser desorption/ionization-time of flight, mass spectrometry (MALDI-TOF) as a tool for differentiating genotypes based on the mass of variant. We have performed multiplex PCR and genotyped 12 SNPs in 758 samples (166 cases and 592 controls). The 12 studied SNPs were chosen with a rationale for their association with multiple cancers in literature.

**Results:**

This is the first study to explore these SNPs with esophageal cancer within the J&K population. Out of 12 SNPs, two SNPs rs12190287 of *TCF21* and rs10046 of *CYP19A1* were significantly associated with esophageal cancer with Odds Ratio (OR) 1.412 (1.09–1.8 at 95% CI, *p* = 0.008) and 1.54 (1.21–2.072 at 95% CI, *p* = 0.0007) within the population of Jammu and Kashmir.

**Conclusion:**

We explored 12 SNPs that were found to be associated with multiple cancers in literature with esophageal cancer within the population of J&K. This is the first study to find the relation of these SNPs with ESCC within the studied population. This study explores the relation of genetic and environmental factors with the ESCC susceptibility.

## Background

Esophageal cancer (EC) is the most common type of cancer worldwide but is least studied with poor survival and highly aggressive nature. Various risk factors have been associated with EC which include lifestyle, dietary habits, low socio-economic status, poor oral hygiene, and genetics [1]. According to a survey by GLOBOCAN in 2018 about 572,034 of new cases and 508,585 deaths were reported worldwide about EC. About 70% of cases have been observed in men, and there is a 2–3 fold difference in incidence and mortality rates between the sexes worldwide [2]. In India, the Northeastern states like Assam, Meghalaya, Mizoram, and Nagaland tops the chart about EC, for both men and women. Kashmir valley is another state with the highest incidence of EC [3]. Genetics is one of the major risk factors associated with EC which includes loss and gain of chromosomes, gene amplification, and microsatellite instability [4]. 90% of esophageal cancers are ESCC (esophageal squamous cell carcinoma) and about 5% are EAC (esophageal adenocarcinoma) throughout the world. The remaining 5% include rare malignancies [4]. We attempted to investigate the role of cancer-related genetic variants in ESCC within the population of J&K. J&K is an ignored state owing to its peculiar geographical background and political circumstances. Although the incidence rate of EC is very high, yet the data related to the genetics of ESCC in the studied population is meager. Replication studies are important to enhance the credibility of a study. Genes that have been explored about ESCC within the population of J&K are summarized in the (Supplementary Table 1).

## Methods

### Sampling

A total of 758 individuals (166 cancerous and 592 healthy controls) were recruited for the study after approval from the Institutional Ethical Review Board (IERB) - Shri Mata Vaishno Devi University (SMVDU) under notification number (SMVDU/IERB/16/41). All the patients recruited were not on chemotherapy, radiotherapy, and do not have any other form of cancer. All the control samples recruited were without any family history of ESCC. A 2 ml of a venous blood sample was collected. A well-written consent was taken from both cases and controls (Supplementary file 1). The clinical parameters of both cases and controls are provided in Supplementary Table 2.

### DNA isolation

The genomic DNA was isolated by using the phenol-chloroform method [5] and the Qiagen DNA isolation Kit (Catalogue No. 51206).

The quality of the genomic DNA was checked by agarose gel electrophoresis (Bio-Rad Gel Doc™ EZ imager) and quantification was performed using Eppendrof’s BioSpectrometer™ at wavelength 260 nm and 280 nm. The amount of DNA was calculated using the following formula, DNA μg/ml = OD at 260 nm x 50X Dilution factor. The ratio at 260/280 was taken as criteria to check the purity of DNA.

### Genotyping

Agena MassARRAY platform was used for SNP genotyping. It is a robust tool with high accuracy and is cost-effective as it involves multiplex PCR [6]. Genotyping was performed in the Central MassARRAY Analyzer facility at Shri Mata Vaishno Devi University. Agena Design Suite v2.0 was used to design-forward, reverse, and single base extension primer (customized) (Supplementary Table 3). Multiplex PCR was used to amplify the targeted region. 1 μl volume of genomic DNA (10 ng/μl concentration) was loaded in 384 well PCR plates which were dried at 85 °C for 10 min. After drying, the reaction mixture was prepared. The reaction mixture includes dNTPs, primer pool (forward and reverse primers pool), reaction buffers, and DNA polymerase. After the first PCR is done, the reaction mixture was treated with shrimp alkaline phosphatase (SAP), the multiplex PCR reaction is then performed with modified ddNTPs and primers (pooled single extension primers). PCR conditions were used using the Gabriel et.al 2009 protocol [6].

After the final PCR, the product was subjected to resin treatment and then is transferred to 384 well Spectro-CHIP using MassARRAY Nanodispenser. The product is then subjected to MT analyzer and the data obtained is analyzed by pre-installed Typer Analyzer v4.0. The genotyping results were replicated in 10% of random samples and the concordance rate was 98.6%. In the reaction of 384 well plates, one positive and one negative control was added for quality check.

### Genotyping quality control

SNPs with a call rate above 90% were only included for further the statistical analysis. All the 12 SNPs were following HWE (Hardy Weinberg Equilibrium) in both cases and controls.

### Statistical analysis

The statistical analysis was done using Plink v1.09 [7] with a maximum of 1000 permutations. All the 12 SNPs were following HWE (Hardy Weinberg Equilibrium). The significance level was calculated based on 3 × 2 chi-square tests for genotype frequencies between ESCC cases and healthy controls. Further logistic regression analysis was also done using SPSS V.23. The analysis was done based on Odds Ratio (OR), confidence interval (CI) and *p*-value as the level of significance from confounding factors like age, gender, and BMI.

GTEx portal was used to determine NES (normalized effect size) value {Low, 2017 #12} and the gene expression of associated SNPs and variant effect prediction (Supplementary Figures 1 and 2). NES value for variants rs10046 is negative and rs12190287 was positive. Both are significantly associated with the esophageal tissue. A positive beta means that the tested allele is associated with an increased expression of that gene; a negative beta means that the tested allele is associated with a reduction in gene expression.

## Results

This is a case-control study that includes 166 ESCC cases and 592 healthy controls belonging to the Northern region of India. The mean age and standard deviation (SD) for cases were 60.4 ± 12.6 and for controls it was 58.4 ± 18.4. A total of 86 males and 80 females (cases) and 192 males and 400 females (controls) were recruited in the study. The BMI which is one of the main risk factors for malignancies was also recorded. The BMI of patients was 21.1 ± 5 and in the case of controls it was 27.6 ± 5.1. In about 68 cases there was metastasis while in 98 cases were non-metastatic. While recording family history, it was observed that about 22 cases had a family history of ESCC, 46 cases did not have a family history and 98 cases were not aware of the history of ESCC in their families. In the present study, two SNPs were showing an increased risk of ESCC within the population. These SNPs are rs12190287 of *TCF21* and rs10046 of *CYP19A1*. In our study, the genetic variant rs12190287 has been evaluated concerning ESCC and it was observed that the variant under study was associated with the higher risk of ESCC within the population of Jammu and Kashmir with OR 1.412 (1.09–1.8, at 95% CI, *p* = 0.008). The genetic variant in *CYP19A1* (Cytochrome P450 family 19 sub-familyA1) rs10046 (C > T) has been associated with a higher risk of ESCC with OR 1.584 (1.21–2.072, at 95% CI, *p* = 0.007) as shown in Table 1.

**Table 1:** Association analyses of the variants with Esophageal Cancer

S.No	SNP	Gene	***p*** value	OR (95% CI)	HWE	OR (95% CI^a^)	***p*** value^a^
1	rs12190287	*TCF21*	0.008	1.4 (1.09–1.8)	0.126	1.3 (1.07–1.8)	0.002
2	rs10046	*CYP19A1*	0.0007	1.5 (1.21–2.0)	0.195	1.8 (1.1–3.2)	0.02
3	rs2735940	*TERT*	0.457	1.1 (0.84–1.45)	0.499	1.1 (0.4–2.4)	0.849
4	rs751402	*ERCC5*	0.597	0.9 (0.68–1.24)	0.847	1.2 (0.7–2.0)	0.535
5	rs2699887	*PIK3CA*	0.063	0.7 (0.49–1.02)	0.117	1.8 (1.0–3.2)	0.03
6	rs3792152	*REV1*	0.094	1.2 (0.96–1.60)	0.412	1.5 (0.8–2.8)	0.170
7	rs10069690	*TERT*	0.994	0.9 (0.72–1.3)	0.709	1.0 (0.5–1.7)	0.952
8	rs2981582	*FGFR2*	0.594	1.1 (0.82–1.39)	0.067	1.3 (0.7–2.1)	0.314
9	rs1695	*GSTP1*	0.723	1.1 (0.77–1.43)	0.908	1.3 (0.7–2.3)	0.269
10	rs251796	*TERF2*	0.299	0.8 (0.65–1.13)	0.864	0.7 (0.4–1.2)	0.270
11	rs2229080	*DCC*	0.199	1.1 (0.91–1.54)	0.113	0.6 (0.2–1.2)	0.173
12	rs1801018	*BCL2*	0.999	1.0 (0.77–1.29)	0.125	0.9 (0.5–1.6)	0.773

^a^Adjusted with age, gender and BMI

In the present study, all the genetic variants were tested individually in a standard way to find its association with the disease. Both significant and non-significant SNPs are equally important for SNP analysis in the case-control study design. The SNP is said to be significantly associated or not associated with the risk of disease based on the *p*-value. If the p-value is less than or equal to a specific threshold (0.05), the SNP is said to be significantly associated with the higher risk of the disease provided OR (odd’s ratio) is above 1 at 95% CI (confidence interval) and if the p-value is greater than 0.05 then the variant is considered to be not associated [8]. The details of both significant and non-significant SNPs have been given in Table 1. All the SNPs summarized in the table below were following HWE. In any genetic association study, HWE is an essential tool to find genotyping errors [9].

## Discussion

Esophageal cancer is a very belligerent type of carcinoma with a very low survival rate. It’s because of its truculent nature, it is least studied worldwide. There are numerous risk factors associated with esophageal carcinoma which include genetic factors and environmental factors [10]. In Asia, ESCC is the most prevalent type of cancer while as in Caucasians there is a higher incidence of EAC. It is because of the different heritage backgrounds that these two populations show different susceptibilities towards esophageal carcinoma [11]. Epidemiological studies have shown that esophageal carcinoma is highly prevalent in the state of Assam located in the eastern region of India [12]. Jammu and Kashmir are one of the high incidence areas of *“Central Asian esophageal cancer belt”.* Central Asian esophageal cancer belt includes northern China, republics of Kazakhstan, Uzbekistan, and Turkmenistan. Kashmir Valley also falls under this high incidence region at the southernmost end [4]. The State of Jammu and Kashmir is geographically divided into three distinct regions Jammu, Kashmir, and Ladakh. They are culturally and ethnically distinct. These regions have historically/geographically married within their communities thus preserving the genetic pool. Though various esophageal carcinoma studies from the Kashmir region have explored demography and genetics but it remains uninvestigated from the Jammu region. There is a major difference between the dietary habits of people from Jammu as compared to people from the Kashmir or the Ladakh region [13]. A detailed questionnaire was designed for the patients in the present study which consisted of information like family history, age, gender, BMI, etc.

In the present study, genetic elucidation among cases and controls was explored. Genetics is an important risk factor associated with esophageal carcinoma. In the SNP analysis we have replicated the newly identified variants (rs12190287 of *TCF21*, rs10046 of *CYP19A1*, rs2735940 of *TERT*, rs751402 of *ERCC5*, rs2699887 of *SLC14A2*, rs3792152 of *REV1*, rs10069690 of *TERT*, rs2981582 of *FGFR2*, rs1695 of *GSTP1*, rs251796 of *TERF2*, rs2229080 of *DCC*, rs1801010 of *BCL2*. Two SNPs that were showing an increased risk of ESCC within the population of our study are rs12190287 of *TCF21* and rs10046 of *CYP19A1*.

***TCF21*** (Transcription factor 21) helps in epithelial differentiation and thus plays a specific role in the differentiation of one or more subsets of epicardial cell types {Copyright© 1996–2018#97}. It is a candidate tumor suppressor located at chromosome 6 and it has been associated with lung, head, and neck cancers. It was found that *TCF21* in gastric carcinoma cells was highly methylated than in the normal adjacent cells. rs12190287 (C > G) of *TCF21* has been studied with Osteosarcoma risk in the Chinese population and was showing a higher risk of Osteosarcoma in the studied population [14].

***CYP19A1*** (Cytochrome P450 family 19 sub-familyA1) gene plays an important role in the biosynthesis of estrogen and it has been associated with the progression of breast cancer in the Chinese population [15]. Several association studies have been done with *CYP19A1* and it was observed that there are enormous inter-population differences. The genetic variant rs10046 (C > T) has been significantly associated with Iranian women, Inuit women, and Xinjiang Uygur women [16].

Both the genetic variants rs12190287 of *TCF21* and rs10046 of *CYP19A1* have been studied in multiple cancers which include breast, lung, neck, and gastric cancer. Our study associated the above genetic variants with ESCC within the population of Jammu and Kashmir. The functional aspects of the variants understudy are yet to be evaluated. A recent study has observed that genetic variants that have been previously associated with one cancer are associated with other cancers too. Cross cancer analysis has been done for the identification of variants to estimate the genetic correlation between them by using the data from GWAS. Substantial evidence has been found which has identified pleiotropy among loci with strong association. A new high throughput study must be used to evaluate the functional implication of the variants [17].

The genes (Table 1) that were not associated with the ESCC in the studied population include ***TERT*(Telomerase Reverse Transcriptase)** which is highly active in cancer cells but shows low or inactivity in normal somatic cells. rs2735940 was significantly associated with a higher risk of ESCC in the Chinese population. This genetic variant has previously been associated with breast cancer in the European population [18, 19]. rs751402 of ***ERCC5 (Excision Repair Cross Complementing Group 5)*** plays an important role in DNA damage and repair and its deficiency can lead to genomic instability and carcinogenesis [20]. There are two genetic variants rs751402 (C > T) and rs2298881 (A/C/T) rs751402 that have been associated with ESCC in the Chinese population and rs2298881 has been associated with other cancers like lung, gastric and laryngeal cancers across the population [21–23]. rs2699887 of ***PIK3CA*(Phosphatidylinositol-4-5-Biphosphate 3-Kinase Catalytic Sub- Unit Alpha)** has been associated with ESCC in the USA (Texan population). rs3792152 of *REV1***(*REV1*, DNA Directed Polymerase)** recruits DNA polymerases involved in translesion synthesis (TLS) of damaged DNA. Genetic variant rs3792152 of *REV1* is associated with breast cancer risk in Thai women [24] and **rs2981582 of*FGFR2*(Fibroblast Growth Factor Receptor 2)** plays a vital role in the cell proliferation, migration, and apoptosis [25]. Genetic variant rs2981582 of *FGFR2* has been associated with breast cancer in different women population (Dutch, Arabic, and West Siberia) [26–28]. rs1695 of ***GSTP1*(Glutathione S-transferase Pi 1)** belongs to a family of enzymes that play an important role in detoxification by catalyzing the conjugation of many hydrophobic and electrophilic compounds. The genetic variant rs1695 of GSTP1 has shown an increased risk of ESCC and EAC in the population of Kashmir Valley [29]. In the present study, though the genetic variant rs1695 of GSTP1 has shown no significant association with ESCC. Similar results were obtained after stratification by race (Caucasian/Asian). Our results were in agreement with the above-mentioned meta-analysis of 52 studies [30]. rs2229080 of ***DCC*(Deleted in Colorectal Carcinoma Netrin1 Receptor)** has been studied in the Chinese population concerning breast cancer and it was found that this variant has been associated with reduced breast cancer risk [31]. The genetic variant rs2229080 (C > G) has been studied in ESCC and gastric cancer patients in Kashmir Valley also and it has been observed that rs2229080 of *DCC* has shown no association with the risk of ESCC. Various studies have shown the role of *DCC* in colorectal carcinoma, gastric, breast, esophageal and prostate cancer [32]. rs1801018 of ***BCL2*(B-cell lymphoma 2)** contributes to programmed cell death and apoptosis and has been studied in ESCC in the Chinese population but there was no significant association observed between the variant and the risk of the disease [33]. All of the above-mentioned SNPs have shown non-significant association with ESCC within the population of J&K in the present study. Although, these variants need to be investigated in the large cohort of population for a conclusive statement.

## Conclusion

The Present study explores the link between genetics, environmental factors, and ESCC. This is the first study to investigate these genetic variants within the population of Jammu and Kashmir about esophageal squamous cell carcinoma. The present study identified important regions of genetic variation associated with risk for the development of the disease. Understanding of these biomarkers will help in elucidating the biological pathways and possible new strategies for identification and prevention of the malignancies. Though it has to be replicated in the large cohort for a conclusive statement but it is an important study that can establish the clinical relevance of novel biomarkers.

## Supplementary information

**Additional file 1: Supplementary Table 1.** Details of the genes selected for the study. **Supplementary Table 2.** Details and clinical features of the cases and controls of J&K Population. **Supplementary Table 3.** SNPs and primers associated.

**Additional file 2: Supplementary Figure 1.** Gene expressions of *CYP19A1* (fig. 1a) and *TCF21* (fig. 1b) in different tissues. Violin plots showing expression of *CYP19A1* and *TCF21* in different tissues which includes esophageal tissue. On the basis of TPM value (Transcript per million) *CYP19A1* is less expressed in esophageal tissue whereas *TCF21* is well expressed in Esophageal tissue and NES value for variants rs10046 of *CYP19A1* is negative and rs12190287of *TCF21* is positive. **Supplementary Figure 2.** Gene location and variant effect prediction. TSS is transcription start site and TES is transcription end site predicted by using The Genotype-Tissue Expression (GTEx) locus. It aims to characterize variation in gene expression levels across individuals and diverse tissues of the human body. Both *CYP19A1* (fig. 2a) and *TCF21* (fig. 2b) has a strong eQTL (expression quantitative trait loci) signal in esophageal tissue.

**Additional file 3: Supplementary file 1.** Datasheet and consent form for sample collection.

## Data Availability

Data and material are available. The datasets generated or analyzed during the current study are not publicly available but are available with the corresponding author and can be provided on reasonable request.

## Abbreviations

BMI: Body Mass Index
CI: Confidence Interval
EC: Esophageal Cancer
ESCC: Esophageal Squamous Cell Carcinoma
HWE: Hardy Weinberg equilibrium
J&K: Jammu and Kashmir
NGS: Next generation sequencing
NES: Normalized effect Size
OR: Odds Ratio
PCR: Polymerase chain reaction
SNP: Single nucleotide polymorphism
SAP: Shrimp alkaline phosphatase
