# Supplementary material for: MassARRAY analysis of twelve cancer related SNPs in esophageal squamous cell carcinoma in J&K, India
Source: BMC Cancer. 2020 Jun 1;20:497. doi: 10.1186/s12885-020-06991-2 (PMC7268327; doi:10.1186/s12885-020-06991-2)
Supplement: Supplementary file 1 — Additional file 1: Supplementary Table 1. Details of the genes selected for the study. Supplementary Table 2. Details and clinical features of the cases and controls of J&K Population. Supplementary Table 3. SNPs and primers associated. [file 12885_2020_6991_MOESM1_ESM.docx]

**Supplementary Table 1: Details of the genes selected for the study**

| **Gene** | **Full name** | **Function** |
| --- | --- | --- |
| ***BCL2*** | B-cell 2 | Apoptosis Regulator |
| ***SLC19A1*** | Solute Carrier Family 19 Member 1 | Transmembrane transporter activity |
| ***CYP19A1*** | Cytochrome P450 Family 19 Subfamily A Member 1 | Catalyzes the formation of aromatic C18 estrogens from C19 androgens. |
| ***FGFR2*** | Fibroblast Growth Factor Receptor 2 | Regulation of cell proliferation, differentiation, migration and apoptosis |
| ***REVI*** | DNA Directed Polymerase | DNA repair |
| ***DCC*** | Deleted in colorectal carcinoma | Tumor suppressor gene. |
| ***TCF21*** | Transcription Factor 21 | Epithelial differentiation |
| ***TERF2*** | Telomeric Repeat Binding Factor 2 | Telomere binding activity and domain organization |
| ***ERCC1*** | Excision Repair 1, Endonuclease Non-Catalytic Subunit | DNA repair |
| ***TERT*** | Telomerase Reverse Transcriptase | Maintenance of telomere ends |
| ***PIK3CA*** | Phosphatidylinositol-4,5-Bisphosphate 3-Kinase Catalytic Subunit Alpha | Serine/threonine kinase activity, oncogene. |
| ***GSTP1*** | Glutathione S-Transferase Pi 1 | Detoxification |
| ***ERCC5*** | Excision Repair 5, Endonuclease Non-Catalytic Subunit | DNA repair |

| **Characteristics** | **Cases** | **Controls** | **p-value** |
| --- | --- | --- | --- |
| Age (±SD) in years | 60.4 ± 5.6 | 58.4 ±8.4 | <0.05 |
| BMI (±SD) in Kg/m^2^ | 21.1 ± 5 | 27.6 ± 5.1 | <0.05 |
| Gender | Males=86  Females=80 | Males=192  Females=400 | - |
| Metastasis | Yes=68  No= 98 | - | - |
| Family history | Yes= 22  No= 48  Unknown= 98 | - | - |

**Supplementary table 2: Details and clinical features of the cases and controls of J&K Population.**

**Supplementary table 3- SNPs and primers associated**

| **S.no.** | **SNP_ID** | **Forward primer** | **Reverse primer** | **UEP_SEQ** |
| --- | --- | --- | --- | --- |
| 1 | rs251796 | ACGTTGGATGAGTGAGCCAAGACCAGAATC | ACGTTGGATGACTTGCCTTTGGGTACTCTG | TGGGACAATCCAGGG |
| 2 | rs751402 | ACGTTGGATGGTATTAGACGGAAACCGAGC | ACGTTGGATGAAACAGCCAGAAGATGTCCC | cGCGGGCCCATTTTTC |
| 3 | rs2735940 | ACGTTGGATGAGGCTTAGGGATCACTAAGG | ACGTTGGATGTGGAGGTTAGCCTCGTCTTG | TTTCTAGAAGAGCGACC |
| 4 | rs2699887 | ACGTTGGATGTGGGACCCGATGCGGTTAGA | ACGTTGGATGATTCCCACCGCACCCGCTA | gGTGAGTAGAGCGCGGA |
| 5 | rs1801018 | ACGTTGGATGGTACTTCATCACTATCTCCC | ACGTTGGATGGTTGCTTTTCCTCTGGGAAG | cctaCCGGTTATCGTACCC |
| 6 | rs3792152 | ACGTTGGATGCCACTCAATAGGAGTTGGAG | ACGTTGGATGGGAATGAAATGGCCTGAACC | gaggTGGAGGTACACCACA |
| 7 | rs2981582 | ACGTTGGATGGCACTCATCGCCACTTAATG | ACGTTGGATGACTGCTGCGGGTTCCTAAAG | CCACTTAATGAACCTGTTTG |
| 8 | rs10046 | ACGTTGGATGGACACTATTGGCAAGGATGG | ACGTTGGATGTGGAACACTAGAGAAGGCTG | cccgGAGAAATGCTCCAGAGT |
| 9 | rs12190287 | ACGTTGGATGTGGAAGGGTATCCTGACATC | ACGTTGGATGATTCTCCAAGGGCTGAGAAC | GCAAATAGACAGGTGGATGAA |
| 10 | rs10069690 | ACGTTGGATGCTGTTTGAAACGGGTTCCTG | ACGTTGGATGTCATCTGAGGAGAGTGTGGG | gtttCACACGGGATCCTCATGCCA |
| 11 | rs2229080 | ACGTTGGATGTCTTGCCCTCTGGAGCATTG | ACGTTGGATGGCTGAGCATCGGTAAATTCC | aaataTGGAGCATTGCAGATCAGC |
| 12 | rs1695 | ACGTTGGATGGCAGATGCTCACATAGTTGG | ACGTTGGATGTGGTGGACATGGTGAATGAC | acacAGTTGGTGTAGATGAGGGAGA |
