# Supplementary material for: MassARRAY analysis of twelve cancer related SNPs in esophageal squamous cell carcinoma in J&K, India
Source: BMC Cancer. 2020 Jun 1;20:497. doi: 10.1186/s12885-020-06991-2 (PMC7268327; doi:10.1186/s12885-020-06991-2)
Supplement: Supplementary file 2 — Additional file 2: Supplementary Figure 1. Gene expressions of CYP19A1 (fig. 1a) and TCF21 (fig. 1b) in different tissues. Violin plots showing expression of CYP19A1 and TCF21 in different tissues which includes esophageal tissue. On the basis of TPM value (Transcript per million) CYP19A1 is less expressed in esophageal tissue whereas TCF21 is well expressed in Esophageal tissue and NES value for variants rs10046 of CYP19A1 is negative and rs12190287of TCF21 is positive. Supplementary Figure 2. Gene location and variant effect prediction. TSS is transcription start site and TES is transcription end site predicted by using The Genotype-Tissue Expression (GTEx) locus. It aims to characterize variation in gene expression levels across individuals and diverse tissues of the human body. Both CYP19A1 (fig. 2a) and TCF21 (fig. 2b) has a strong eQTL (expression quantitative trait loci) signal in esophageal tissue. [file 12885_2020_6991_MOESM2_ESM.docx]

**
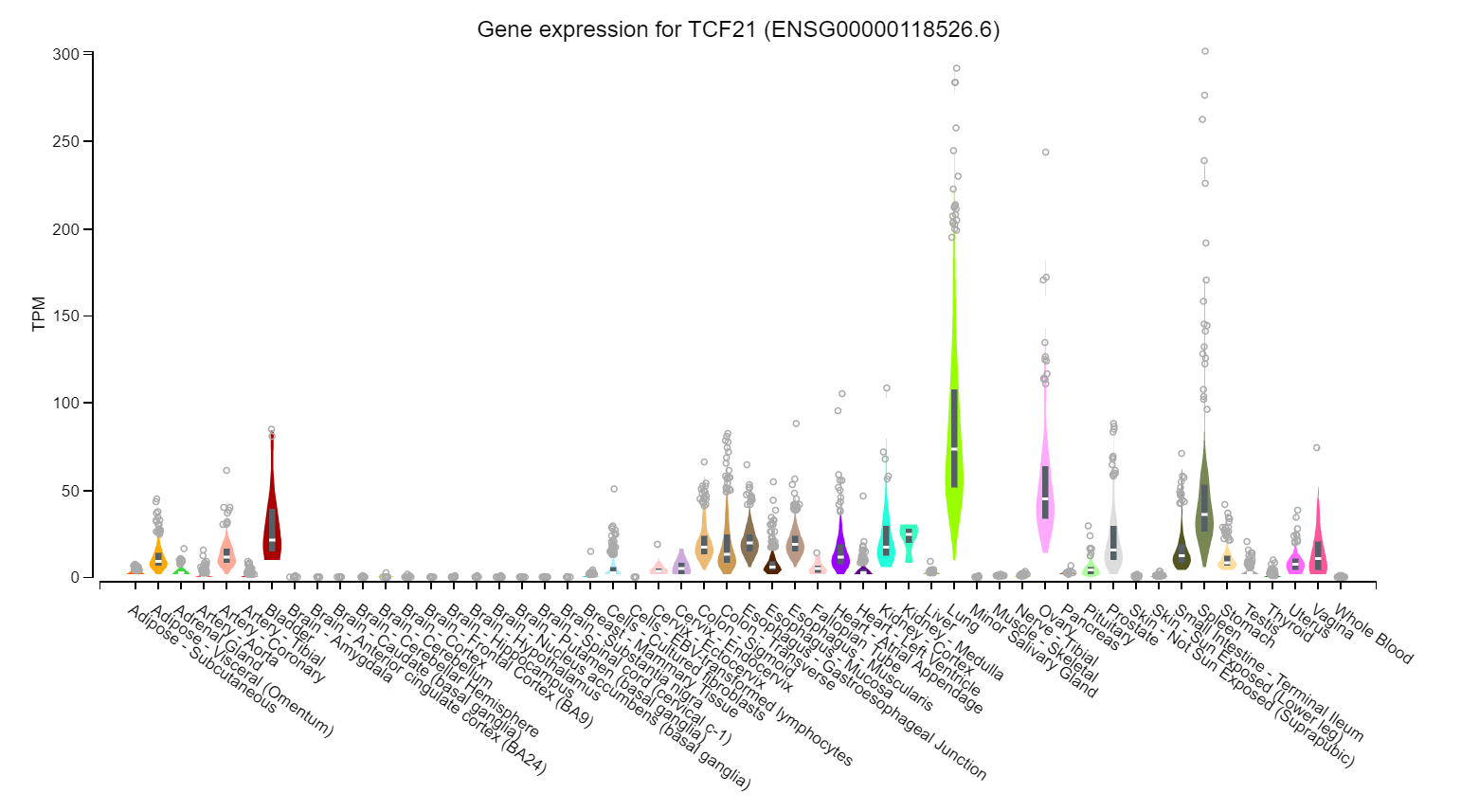

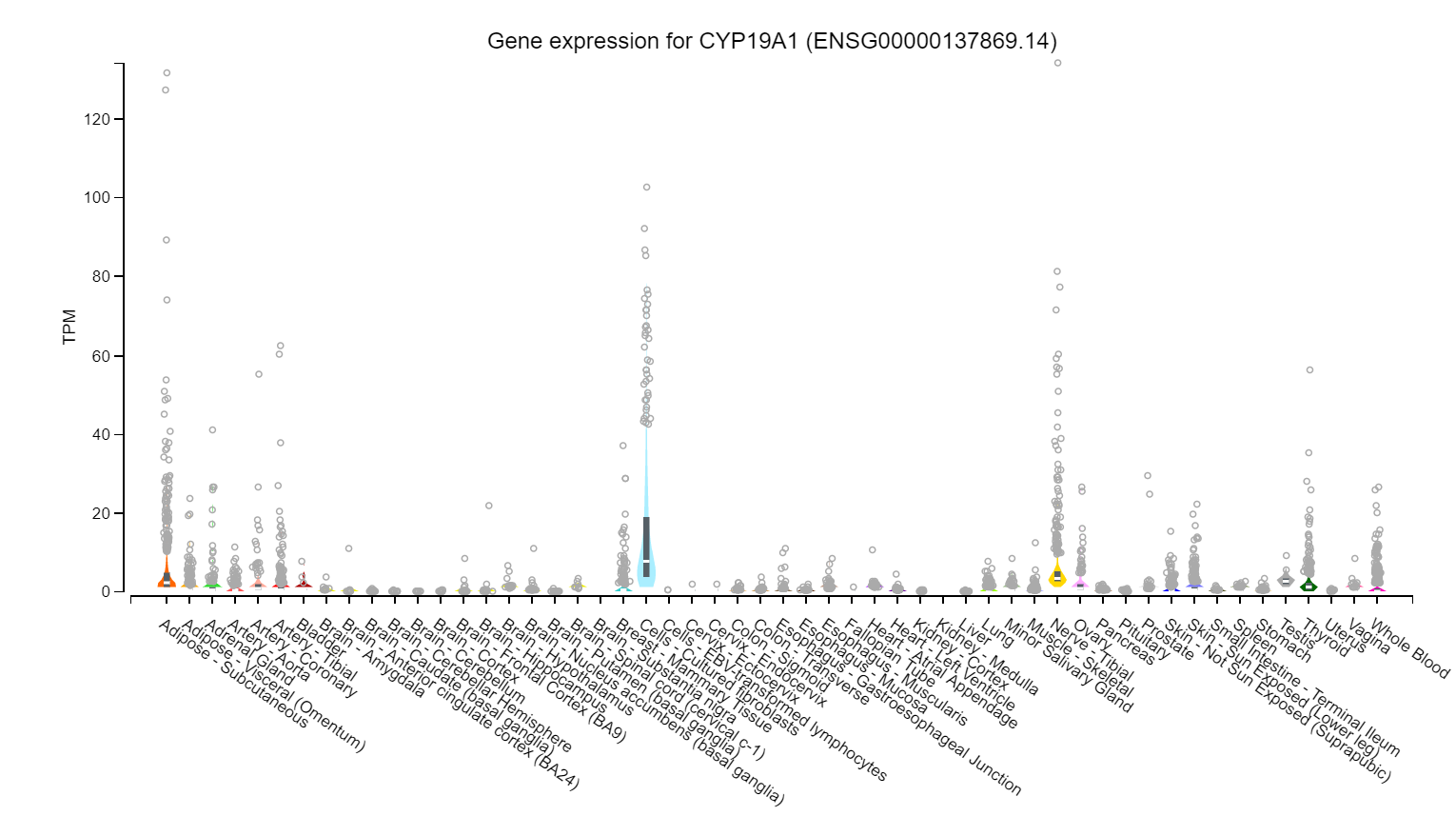
**

**Supplementary Figures**

**(a)**

**(b)**

**Supplementary fig 1 Gene expressions of *CYP19A1* (fig 1a) and *TCF21* (fig 1b) in different tissues.** Violin plots showing expression of *CYP19A1* and *TCF21* in different tissues which includes esophageal tissue. On the basis of TPM value (Transcript per million) *CYP19A1* is less expressed in esophageal tissue whereas *TCF21* is well expressed in Esophageal tissue and NES value for variants rs10046 of *CYP19A1* is negative and rs12190287of *TCF21* is positive.


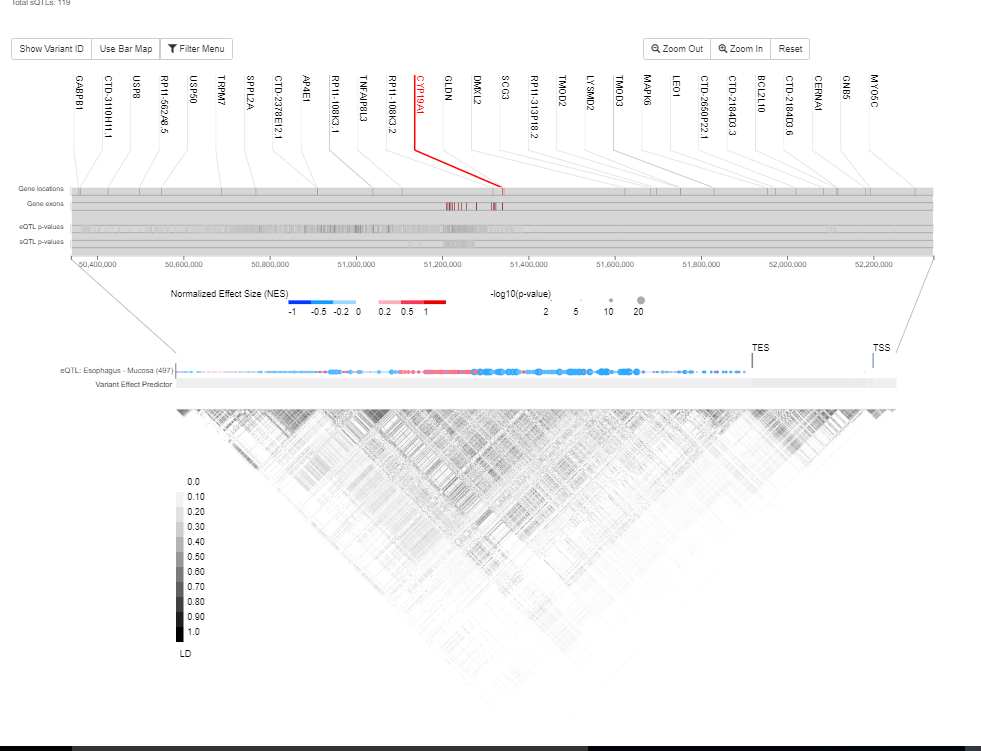

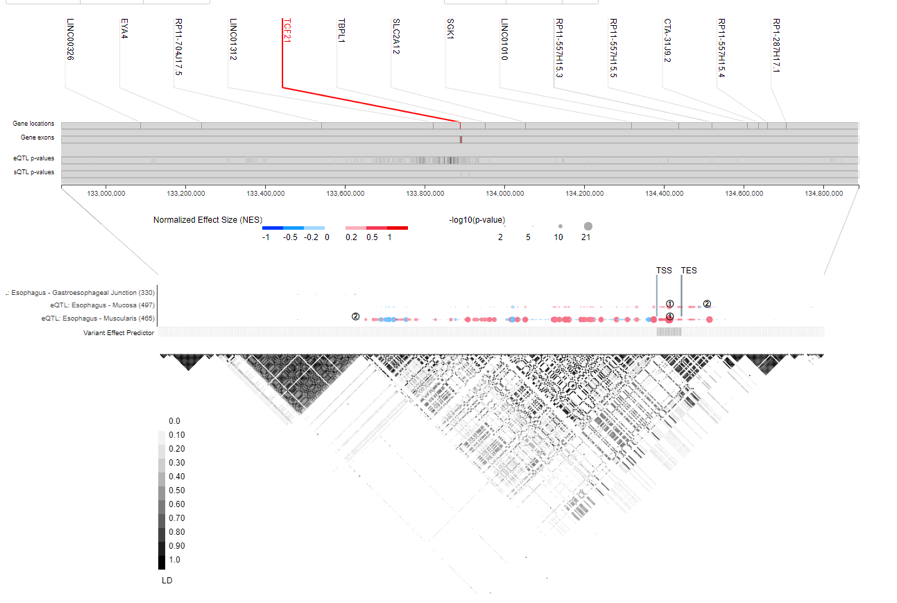


**(b)**

**(a)**

**Supplementary fig 2** Gene location and variant effect prediction. TSS is transcription start site and TES is transcription end site predicted by using The Genotype-Tissue Expression (GTEx) locus. It aims to characterize variation in gene expression levels across individuals and diverse tissues of the human body. Both *CYP19A1* (fig 2a) and *TCF21* (fig 2b) has a strong eQTL (expression quantitative trait loci) signal in esophageal tissue.
