# Supplementary material for: MassARRAY analysis of twelve cancer related SNPs in esophageal squamous cell carcinoma in J&K, India
Source: BMC Cancer. 2020 Jun 1;20:497. doi: 10.1186/s12885-020-06991-2 (PMC7268327; doi:10.1186/s12885-020-06991-2)
Supplement: Supplementary file 3 — Additional file 3: Supplementary file 1. Datasheet and consent form for sample collection. [file 12885_2020_6991_MOESM3_ESM.pdf]

श्री माता वैष्णो देवी विश्वविद्यालय
Shri Mata Vaishno Devi University
Kakryal (Katra) - 182 320
www.SMVDU.net.in

Datasheet and consent form for sample collection

Code: Related codes and Relationship:

Name of the Subject: ______________________________________

Age: ___ Sex: ____Religion: _________Caste/ethical group: ______

Economic status: _______________Place of origin_______________

Height: _________Weight: _______Blood Pressure: _____________

Cancer type: _______________ Age of onset: ____Duration: ______

Therapy (Chemotherapy/Radiotherapy): ________________________

Drug: ______________Family history of any cancer______________

Any other disease: _____________Food Habits: _________________

Signature of Patient/Subject: ___________Signature of PI/Co-PI_____

(رضامندی فارم)

اس تحقیق کے فائدے اور نقصانات مجھے اچھی طرح سے سمجھائے گئے ہیں، جس میں میری شرکت کی توقع ہے، جس کے خاطر مجھے اپنے خون/تھوک/بال کا نمونہ عطیہ کرنا ہوگا۔ میں اپنی مرضی سے، محقق کے کسی دباؤ کے بغیر: (i) اس تحقیق میں حصّہ لینے سے اتفاق کرتا ہوں۔ (ii) اپنا اور اپنے بچوں کا 5ml (۵ ml) خون عطیہ کرنے پہ آمادہ ہوں۔ میری رضامندی میرے ذاتی معلومات افشا کرنے کے لئے نہیں ہے۔ میرے نمونے پہ کی جانے والی تحقیقات سے حاصل کردہ کسی طرح کی ذاتی معلومات افشا کرنے کے لئے مزید رضامندی حاصل کی جانی چاہیئے۔ مجھے بتایا گیا ہے کہ SMVDU اور محققین (PI .... اور اس/ان کے ساتھی) مجھ سے اجازت کے قبل میرے نمونے کی بنیاد پہ کی گئی تحقیق سے فوائد نہیں ہونگے۔

دستخط: (نام)) گواہ: (نام) (محقق: (نام) جگہ:
تاریخ:

CONSENT FORM (for the subject/ patient)

The advantages and disadvantages of the research in which I am expected to participate, for which I have to donate blood/ sputum/hair sample has been explained to me. I willingly, under no pressure from the researcher (i) agree to take part in this research, and agree to participate in all investigations which will help acquire knowledge for the benefit of the mankind and (ii) agree to donate mine and my children's 5 ml blood. My consent is explicitly not for disclosing any personal information. For disclosing any such personal information obtained from the investigations conducted on my samples, further consent should be obtained. I have been informed that SMVDU and the researchers (PI .... and her/his colleagues) will take my prior consent before they draw benefits from research based on my samples.

Signatures Subject/patient (Name:)   Witness (Name:)   Principle Investigator (Name:)   **Place:** **Date:**

Parent's Signature (in case the subject is a minor):

सहमति पत्र

मुझे शोधकर्ता द्वारा जिस उद्देश्य के लिए मुझे शोधकार्य में भाग लेना है रक्तदान और उत्तकदान करना है उसके फायदे और नुकसान बता दिये गये हैं। मैं बिना किसी दबाव के अपनी इच्छानुसारः (१) इस शोधकार्य में भाग लेने के लिए सहमत हूँ इस शोधकार्य के लिए सभी प्रकार के परीक्षण जो मानव जाति के कल्याण के लिए ज्ञान प्रदान करते हैं के लिए सहमत हूँ (२) इस शोधकार्य के लिए अपने या अपने बच्चों का ५ मि ली रक्तदान कर रहा हूँ। मेरी सहमति प्रत्यक्ष रुप से किसी भी व्यक्तिगत जानकारी को सार्वजनिक करने के लिए नही है एवं मेरे नमूनों से प्राप्त किसी व्यक्तिगत जानकारी को सार्वजनिक करने के लिए मेरी अगली अनुमति अनिवार्य है। मुझे यह जानकारी दे दी गयी है श्री माता वैष्णो देवी विश्वविद्यालय और इसके प्रधान शोधकर्ता एवं इनके सहयोगी किसी भी लाभ के कार्य से पहले जो मेरे रक्त या उत्तक नमूनों की जानकारी पर आधारित है मेरी पुनः अनुमति लेंगे।

सहमतिकर्ता के हस्ताक्षर (नाम -)   गवाह के हस्ताक्षर (नाम -)   प्रधान शोधकर्ता के हस्ताक्षर (नाम -)   दिनाङ्क- स्थान-

संरक्षक के हस्ताक्षर (सहमतिकर्ता यदि बालक हो):
